# Convergence between Wnt-β-catenin and EGFR signaling in cancer

**DOI:** 10.1186/1476-4598-9-236

**Published:** 2010-09-09

**Authors:** Tianhui Hu, Cunxi Li

**Affiliations:** 1 Cancer Research Center, Xiamen University Medical College, Xiamen 361005, China; 2 Department of Medicine, Vanderbilt University School of Medicine, Nashville, TN 37232, USA

## Abstract

Wnt and EGFR signaling play key roles in embryonic development and cell proliferation. It is well documented that dysregulation of these two pathways often leads to tumorigenesis with poor prognosis. However, the possible crosstalk between the two pathways in cancer development is largely unknown. Although some reports show that EGFR might antagonize Wnt signaling during development in Drosophila, an increasing body of evidence indicates that Wnt and EGFR signaling crosstalk and transactivate one another in development and cancer. This review summarizes recent studies on the crosstalk between Wnt and EGFR signaling in cancers and points out several possible convergence points. Wnt ligands can activate EGFR signaling through their 7-transmembrane domain receptor Frizzled while EGFR can activate β-catenin via receptor tyrosine kinase-PI3K/Akt pathway; EGFR has been shown to form a complex with β-catenin and increase the invasion and metastasis of cancer cells. NKD2, a Wnt antagonist by interacting with Dishevelled, also escorts TGFα-containing exocytic vesicles to the basolateral membrane of polarized epithelial cells. Down-regulation of NKD2 causes Wnt activation and TGFα misdelivery, suggesting its functions in cell homeostasis and prevention of tumorigenesis.

## 1. Introduction

Tumorigenesis is a complex process requiring the accumulated alteration of multiple genes and pathways. In particular, human colorectal cancers represent a paradigm for the molecular and genetic mechanisms underlying tumor formation and progression [1]. More than 80% of colonic adenomas and carcinomas have mutations in Adenomatous polyposis coli (APC) gene, and loss of APC function results in constitutive activation of Wnt signaling [2]. EGFR signaling plays critical roles in the genesis of adenomas and maintenance of carcinomas during intestinal tumorigenesis [3]. Overexpression of EGFR is found in more than 1/3 of the epithelial carcinomas and may be linked to an advanced stage [4] or may predict a potential metastatic risk in the colon [5], indicating the importance of EGFR signaling in colorectal cancer development. It has been well documented that Wnt and EGFR signaling pathways are closely linked with cancers, but the possible convergence between them is largely unknown. Here we summarize the current studies on the correlation between Wnt and EGFR signaling pathways.

## 2. Wnt Signaling Pathway in Cancers

Wnt signaling plays central role in embryogenesis and human diseases including cancers. Wnt signals can be either transduced to the canonical Wnt pathway for cell fate determination or to the non-canonical Wnt pathway for the control of tissue polarity and cell movement. Canonical and non-canonical Wnt pathways can be differentially activated by different Wnt ligands (Wnt1, 2, 3, 3A, 8A, 8B, 10A and 10B for canonical Wnt pathway and Wnt4, 5A, 5B, 6, 7A, and 7B for non-canonical Wnt pathway) [6]. Wnt11 has recently been shown to be the activator of both canonical and non-canonical Wnt pathways [7]. Dishevelled, the hub of Wnt signaling, can mediate canonical and non-canonical Wnt signaling by binding to different proteins via its different functional domains [8]. Numerous studies have shown that dysregulation of the canonical Wnt pathway leads to cancer development and progression. The non-canonical Wnt pathway has been thought to play key roles in embryonic development and cell polarity. However, in recent years, emerging data indicate that non-canonical Wnt signaling also promotes the invasiveness and metastasis of different cancers [9].

### 2.1 Canonical Wnt signaling pathway in cancers

Canonical Wnt signals are transduced through Frizzled/LRP5/6 complex to stabilize β-catenin by preventing its phosphorylation-dependent degradation and to activate downstream targets. Canonical Wnt signaling is closely related with many cancers [10]. Mutations in APC gene have been identified as one of the basis for colorectal cancer development. In ovarian tumors, APC was found to be absent in all tumors with nuclear β-catenin staining [11]. Mutations in β-catenin, which abrogate its regulation by APC, represent an alternative route to Wnt activation and a basis for cancer development. Axin, one of the important regulators of the Wnt pathway, is also mutated in a variety of human cancers [12]. T-cell-specific transcription factor 4, a β-catenin binding protein, is mutated in nearly half of the micro satellite instable colon cancers [13]. Other Wnt factors are also involved in cancer development. NKD1, a negative regulator, has been shown mutated in colorectal cancers [14]. PP2A, another component of the Wnt pathway, is found to have mutations in its regulatory subunit in some cancers [15]. In summary, oncogenic deregulation of the Wnt signaling pathway is a causal factor in the initiation of cancer in a diverse range of tissues. Due to the close relationship between the canonical Wnt pathway and cancers, inhibition of Wnt activity has become a goal for therapeutic prevention.

### 2.2 Non-canonical Wnt signaling pathway in cancers

The non-canonical Wnt signaling pathway is often referred to as the Planar Cell Polarity (PCP) pathway and the Wnt/Ca^2+ ^pathway. Human Wnt5A, Wnt5B and Wnt11 are non-canonical Wnt ligands transducing PCP signals through FZD3 or FZD6 receptors. Upon ligand binding, non-canonical Wnt signaling controls tissue polarity and cell movement through the activation of RhoA, c-Jun N-terminal kinase (JNK), and nemo-like kinase (NLK) signaling cascades. The well-known role of these pathways is the regulation of morphogenetic processes. However, recently more and more data indicate that components of these pathways might also promote the invasiveness and malignant progression of cancers. There is strong evidence that Wnt5A, the non-canonical Wnt ligand, is involved in cancer progression [16]. Although there are still arguments whether it is a tumor suppressor or promoter, Wnt5A overexpression has been found to be associated with aggressive tumor biology and poor prognosis [17,18]. In vitro studies also confirm that Wnt5A activity increases melanoma invasiveness and that the activity is independent of β-catenin [19]. In colorectal cancers, studies show that non-canonical Wnt signaling antagonizes β-catenin dependent transcription [20], suggesting an anti-oncogenic effect of non-canonical Wnt signaling. However, VANGL1, a PCP pathway protein, has been shown to promote the metastasis of colon cancer. In summary, although in part still controversial, the fact that non-canonical Wnt signaling functions either as a tumor suppressor or promoter is obviously dependent on the individual intra- and intercellular context.

Although canonical and non-canonical Wnt pathways act differently in tumorigenesis, they might also crosstalk in some cancers. In hepatocellular carcinomas, canonical and non-canonical Wnt pathways might have complementary roles, where the canonical signaling contributes to tumor initiation, and non-canonical signaling to tumor progression [21].

## 3. EGFR Signaling Pathway in Cancers

EGFR is a transmembrane receptor of the four ErbB family members, and seven different ligands can selectively bind to each receptor [22]. The majority of human epithelial cancers are marked by the activation of EGFR, and it was the first growth factor receptor to be proposed as a target for cancer therapy. Dysregulation of EGFR is often observed in association with carcinogenesis, which can be caused by receptor overexpression, mutations or deletions [23]. Overexpression of EGFR or ErbB2 leads to the in vitro transformation of NIH-3T3 cells [24,25]. Overexpression of the EGFR ligand TGFα also results in transformation of Rat-1 and NRK cells [26,27]. Blockade of EGFR results in inhibition of growth in several human carcinoma cell lines [28]. Overexpression of EGFR and its family members have been found in the majority of human cancers. On average, 50% to 70% of lung, colon and breast cancers have EGFR and ErbB3 overexpression [28]. Cancer patients with EGFR overexpression often have a worse prognosis. For example, among non-small cell lung cancer (NSCLC) patients, 60% have been reported with EGFR overexpression and a poor prognosis (the median survival time is around 4-5 months) [29]. Additional study shows that co-expression of different ErbB receptors is usually associated with a worse prognosis compared to single receptor overexpression in cancers [30]. In addition to being overexpressed, EGFR is also found to be mutated in different cancers. An in-frame deletion of exon 2-7 of EGFR is frequently detected in glioblastoma, which encodes a constitutively active EGFR protein [31]. Mutations in EGFR are often correlated with EGFR activation and resistance to anti-EGFR treatment. An acquired T790M mutation was found in a NSLCL patient resistant to the drug Gefitnib by increasing the affinity to ATP [32,33]. Tumors with both T790M and L858R are more aggressive [34]. A V665M mutation in the juxtamembrane region of EGFR promotes cellular transformation and tumorigenesis, suggesting this region might be an activation domain [35].

In summary, ErbB receptors and their ligands form a network and are closely involved in cancer development and progression. Overexpression and constitutive activation of EGFR in cancers are often related with a poor prognosis.

## 4. The convergence between Wnt and EGFR signaling in cancers

Cancer development is a complex progress in which many signaling pathways are involved. Cross-communication between different pathways allows the integration of the great diversity of stimuli. Wnt and EGFR pathways have been reported to closely interact in tumorigenesis, but how they cross-talk and co-activate tumor progression remains an unanswered, interesting topic.

### 4.1. Wnt and EGFR signaling in cell proliferation and embryonic development

Signaling by EGFR plays a critical role in the segmental patterning of the ventral larval cuticle in Drosophila. Bienz and coworkers showed that EGFR signaling antagonizes Wnt signaling in the larval cuticle [36]. A following study showed that EGFR/rolled MAP kinase signaling antagonizes Wnt signaling in the Drosophila eye [37]. Phyllopod, a transcriptional target of the EGFR pathway, blocks Wingless and Notch signaling in Drosophila [38]. However, there are also other reports showing that Wnt and EGF pathways act together to establish planar cell polarity in the Drosophila eye or induce C. elegans male hook development [39,40]. In NIH3T3 cells, Wnt3a stimulates cell proliferation and motility via EGFR-mediated ERK pathway activation [41]. These results may suggest that during cell proliferation or development in different animal models or organs, Wnt and EGFR signaling might crosstalk differently.

### 4.2. Wnt and EGFR signaling pathways synergistically induce tumorigenesis

David Lee and coworkers found that in WAP-TGFα mice the latency of mammary tumorigenesis was greatly reduced. When they co-transfected MMTV to induce the expression of Wnt1 and Wnt3, the latency was further reduced. These results indicate a collaboration between Wnt and EGFR signaling pathways in mammary gland tumorigenesis and suggest a convergence between their ligands (Wnt3 and TGFα ) [42]. Prostaglandin E2 is often implicated in promoting colon cancer development. Studies indicate that prostaglandin E2 (PGE2), a product of cyclooxygenase-2 (Cox-2) activity, promotes tumor growth by activating EGFR [43] or β-catenin [44]. In Min/+ tumors, in which Wnt activity is high, PGE2 is up-regulated and transactivates EGFR [45]. Since both Wnt and EGFR signaling can act on β-catenin, it is possible that Wnt and EGFR pathways converge on β-catenin. Subsequent work indicated a direct interaction between β-catenin and EGFR/ErbB2 heterodimers in mammary gland tumors [46].

It is likely that TGFα and Wnts activate different target genes and may interact cooperatively to promote tumorigenesis. In non-small cell lung cancers, EGFR mutations were significantly associated with a good prognosis in patients that had tumors with unmethylated Wnt antagonist genes, suggesting synchronous alterations of Wnt and EGFR signaling pathways are involved [47]. In intestinal tumor cells, APC and KRAS, a downstream target of EGFR signaling, act synergistically in enhancing Wnt signaling, tumor formation and progression [48]. In breast cancers, Wnt pathway is rarely mutated. However, an extracellular inhibitor of Wnt signaling, secreted Frizzled-related protein 1 (sFRP1), which competes with Frizzled receptors for ligand binding, is often down-regulated, resulting in Wnt deregulation, and those patients usually have a poor prognosis [49,50]. In sFRP1 knockdown breast cancer cell lines, EGFR is transactivated [51], indicating a synergistic effect of Wnt and EGFR signaling in breast cancer development. It should be noticed that tumors arising from activation of ErbB and Wnt pathways in transgenic mice display distinct pathologies [52], suggesting some independency between Wnt and EGFR mediated tumorigenesis.

### 4.3. Crosstalk between Wnt and EGFR pathways in cancers

Crosstalk between Wnt and EGFR has been identified in some tumors. In breast cancers, Wnt overexpression activates signaling via EGFR [53,54]. In HC11 mammary epithelial cells, constitutive expression of Wnt1 and Wnt5a accompanies activation of EGFR and MAPK. Inhibition of EGFR kinase activity and addition of sFRP1 both prevent this effect. TGFα and other EGFR ligands are not induced by Wnt-1 or Wnt-5a, but addition of metalloproteinase inhibitors blocks the stimulation of EGFR and ERK phosphorylation. Thus, Wnt activation of EGFR is apparently mediated by an increase in the availability of EGFR ligands [55]. Further studies showed that in breast cancers, Wnt1 transactivates EGFR, implying that constitutive Wnt signaling might impact not only the canonical pathway but also EGFR activity by augmenting ligand availability [51]. In liver-specific non-mutated β-catenin-overexpressing transgenic mice, EGFR seems to be a direct target of the activated Wnt signaling pathway, and EGFR activation might contribute to some mitogenic effect of increased β-catenin in the liver [56]. In NSCLC, there is a positive correlation between activated EGFR mutation and nuclear accumulation of β-catenin [47]. All of these results suggest a close correlation between Wnt and EGFR signal pathways in cancers.

Many studies indicate that Wnt and EGFR signaling crosstalk via receptor tyrosine kinase pathways. EGFR mediated PI3K/Akt activation promotes β-catenin transactivation and tumor cell invasion, suggesting that EGFR activation transactivate β-catenin activity via receptor tyrosine kinase pathways in tumor cells [57-60]. In breast cancers, upregulation of Wnt-1 induces EGFR and Erk 1/2 MAPK activation [53]. In APC deficient mice, Wnt activity causes EGFR/PI3K/Akt activation [45].

### 4.4. Possible convergent points between Wnt and EGFR pathways

#### Frizzled

EGFR can be transactivated upon G protein coupled receptor (GPCR) stimulation. This transactivation involves proHB-EGF and a metalloproteinase activity that is rapidly induced upon GPCR-ligand interaction [61,62]. The Frizzled receptors through which Wnts act are 7-transmembrane domain receptors that are structurally related to other families of G-protein-coupled receptors. When Wnt1 and Wnt5a bind to Frizzled, it transactivates EGFR signaling by matrix metalloproteinase-mediated release of soluble EGFR ligands [55]. All these data suggest that Frizzled is a convergence point of Wnt and EGFR pathways.

#### β-catenin

Studies show that EGF treatment of human breast cancer cell lines MDA-MB-468 can induce a strong tyrosine phosphorylation of β-catenin [63], that blocks the interaction between β-catenin and E-cadherin and increases the invasiveness and metastatic potential of cancer cells [64,65]. Chronic activation of EGFR induces transcriptional down-regulation of caveolin-1, which in turn enhances β-catenin-TCF/LEF-1 transcriptional activity in a GSK-3β-independent manner [57]. Using the murine mammary tumor virus (MMTV)-Wnt-1 transgenic model of mammary carcinoma, Schroeder and his colleagues have identified an unvarying association between β-catenin and epidermal growth factor receptor/c-Neu (ErbB1/ErbB2) heterodimers in mammary gland tumors, indicating a requirement for ErbB signaling in Wnt-mediated tumorigenesis [46]. Studies also show that EGFR activation could induce nuclear accumulation of β-catenin via PI3K/Akt pathway in prostate cells [59,60]. In liver-specific non-mutated β-catenin-overexpressing transgenic mice, EGFR seems to be a direct target of the pathway, and EGFR activation might contribute toward some mitogenic effects of increased β-catenin in the liver [56]. All of these studies indicate that EGFR and β-catenin may be cooperating in tumorigenesis and that β-catenin might be a convergent point between EGFR and Wnt signaling in cancer development.

#### NKD2

NKD1 and NKD2 are two mammalian orthologs of Drosophila Naked cuticle and have been shown to negatively regulate canonical Wnt signaling through an interaction with Dishevelled (Dvl) [66-68]. In zebrafish, NKD1 and NKD2 antagonize both canonical and non-canonical Wnt signaling [69]. Katoh investigated the expression of NKD1 and NKD2 in human cancer cell lines and primary gastric cancer. He found that NKD1 was up-regulated in the colorectal cancer cell line SW480, gastric cancer cell line TMK1, and pancreatic cancer cell line Hs700T, while NKD2 was up-regulated in the gastric cancer cell line MKN45, pancreatic cancer cell line BxPC-3, and esophageal cancer cell lines TE6, and TE13, indicating NKD1 and NKD2 might be candidate tumor suppressors [70]. NKD2, but not NKD1, also interacts with the cytoplasmic C-terminal fragment of a Golgi-processed form of TGFα, coats TGFα-containing exocytic vesicles, and escorts those vesicles to the basolateral membrane of polarized epithelial cells in a myristoylation-dependent manner [71]. NKD2 is an intrinsically unstructured protein and acts as a cargo recognition and targeting protein to ensure proper delivery and fusion of TGFα-containing exocytic vesicles [72-74]. NKD2 can be stabilized by TGFα[75] but down-regulated by Dishevelled in HEK293T cells [76]. The above results indicate that NKD2 might be a regulator of both Wnt and EGFR signal pathways by regulation of TGFα delivery and Dishevelled stabilization. Although we have never observed a tertiary complex between NKD2, TGFα and Dishevelled, our results show that NKD2 forms a mutual degradation complex with Dvl-1 [76], and that TGFα stabilizes NKD2 by suppressing the binding between NKD2 and its ubiquitin ligase AO7 [75]. Based on our observations, we propose a model for the regulatory role of NKD2 in Wnt and EGFR signaling pathways: NKD2 binds to TGFα and escorts it to the plasma membrane, where TGFα gets released, and then NKD2 binds to Dvl-1 and targets each other for mutual degradation. NKD2 might be an important convergent point between Wnt and EGFR pathways to maintain the epithelial cell homeostasis.

## 5. Conclusion

**Figure 1:**
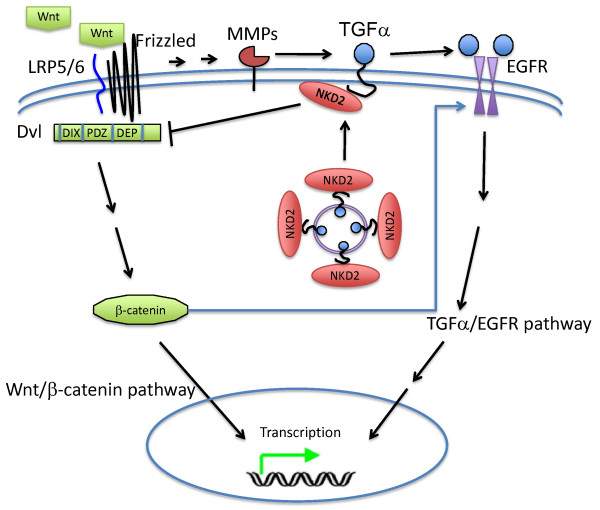
**Convergence between Wnt and EGFR pathways**. Wnt binds to Frizzled. Frizzled transactivates EGFR signaling by matrix metalloproteinase-mediated release of soluble EGFR ligands. Upon activation, EGFR could transactivate β-catenin, possibly through receptor tyrosine kinase-PI3K/Akt pathway, and β-catenin might also form heterodimer with EGFR and activate EGFR pathway. NKD2 binds to TGFα and escorts it to the plasma membrane, where TGFα gets released, and then NKD2 binds to Dvl-1 and targets it for mutual degradation, thus maintains the epithelial cell homeostasis.

The crosstalks between Wnt and EGFR are summarized in Fig. 1.

Both Wnt and EGFR signaling are closely related with tumorigenesis. In recent years a considerable body of evidence shows that Wnt and EGFR crosstalk with each other in cancer development. Addition of Wnt ligands transactivates EGFR signaling, possibly through Frizzled and its downstream partners. EGFR can form a complex with β-catenin and further activate Wnt pathway. In cancers, mutations or dysregulation in the Wnt pathway often induce EGFR activation. This review also points out several possible convergence points between Wnt and EGFR signaling, such as Frizzled, β-catenin and NKD2. Tight regulation of those proteins maintains the homeostasis and prevents from tumorigenesis. Further studies will surely disclose more convergence points between Wnt and EGFR signaling.

Mutations in key proteins of Wnt and EGFR pathways have been found in most of the cancers. 80% of colon cancers have APC mutations [2] and 50-70% of breast, colon and lung cancers have EGFR and ErB3 mutations [28]. However, what percentage of conincidence of mutations in both EGFR and Wnt pathways in those patients, remain a very important and interesting topic. To elucidate this question will surely help further to understand the roles of Wnt and EGFR convergence in cancer development.

## Competing interests

The authors declare that they have no competing interests.

## Authors' contributions

TH wrote and drafted the manuscripts. LC revised the manuscript critically. All authors read and approved the final manuscript
